# The HIV-Brazil Cohort Study: Design, Methods and Participant Characteristics

**DOI:** 10.1371/journal.pone.0095673

**Published:** 2014-05-01

**Authors:** Alexandre Grangeiro, Maria Mercedes Escuder, Alex Jones Flores Cassanote, Rosa Alencar Souza, Artur O. Kalichman, Valdiléa Veloso, Maria Letícia Rodrigues Ikeda, Nêmora Tregnago Barcellos, Carlos Brites, Unai Tupinanbás, Noaldo O. Lucena, Carlos Lima da Silva, Heloisa Ramos Lacerda, Beatriz Grinsztejn, Euclides Ayres Castilho

**Affiliations:** 1 Department of Preventive Medicine, University of São Paulo School of Medicine, São Paulo, Brazil; 2 Health Institute, São Paulo State Department of Health, São Paulo, Brazil; 3 Postgraduate Program in Infectious and Parasitic Diseases, University of São Paulo School of Medicine, São Paulo, Brazil; 4 STD/AIDS Referral and Training Centre, São Paulo State Department of Health, São Paulo, Brazil; 5 Evandro Chagas Clinical Research Institute, Oswaldo Cruz Foundation, Rio de Janeiro, Brazil; 6 Care and Treatment Clinic of the Partenon Sanatorium, Rio Grande do Sul State Department of Health, Porto Alegre, Brazil; 7 Edgar Santos University Hospital Complex, Federal University of Bahia, Salvador, Brazil; 8 Medical School, Federal University of Minas Gerais, Belo Horizonte, Brazil; 9 Tropical Medicine Foundation, Amazonas State Department of Health, Manaus, Brazil; 10 State Centre for Diagnosis, Treatment and Research, Bahia State Department of Health, Salvador, Brazil; 11 Federal University of Pernambuco, Recife, Brazil; Faculty of Health Sciences, University of Cape Town, South Africa

## Abstract

**Background:**

The HIV-Brazil Cohort Study was established to analyze the effectiveness of combination antiretroviral therapy (cART) and the impact of this treatment on morbidity, quality of life (QOL) and mortality. The study design, patients’ profiles and characteristics of cART initiation between 2003 and 2010 were described.

**Methodology/Principal Findings:**

Since 2003, the HIV-Brazil Cohort has been following HIV-infected adults receiving cART at 26 public health care facilities, using routine clinical care data and self-reported QOL questionnaires. When not otherwise available, data are obtained from national information systems. The main outcomes of interest are diseases related or unrelated to HIV; suppression of viral replication; adverse events; virological, clinical and immunological failures; changes in the cART; and mortality. For the 5,061 patients who started cART between 2003 and 2010, the median follow-up time was 4.1 years (IQR 2.2–5.9 years) with an 83.4% retention rate. Patient profiles were characterized by a predominance of men (male/female ratio 1.7∶1), with a mean age of 36.9 years (SD 9.9 years); 55.2% had been infected with HIV via heterosexual contact. The majority of patients (53.4%) initiated cART with a CD4^+^ T-cell count ≤200 cells/mm^3^. The medications most often used in the various treatment regimens were efavirenz (59.7%) and lopinavir/ritonavir (18.2%). The proportion of individuals achieving viral suppression within the first 12 months of cART use was 77.4% (95% CI 76.1–78.6). Nearly half (45.4%) of the patients presented HIV-related clinical manifestations after starting cART, and the AIDS mortality rate was 13.9 per 1,000 person-years.

**Conclusions/Significance:**

Results from cART use in the daily practice of health services remain relatively unknown in low- and middle-income countries, and studies with the characteristics of the HIV-Brazil Cohort contribute to minimizing these shortcomings, given its scope and patient profile, which is similar to that of the AIDS epidemic in the country.

## Introduction

In 1996, Brazil adopted a policy of universal access to combination antiretroviral therapy (cART), which was free of charge to human immunodeficiency virus (HIV)-infected individuals. Since then, immunological and virological treatment monitoring, as well as genotype-resistance testing for treatment failure management, have been incorporated into that policy. As of 2012, approximately 217,000 patients in Brazil were being treated with first-, second- or third-line antiretroviral therapy, including newer antiretroviral options for salvage therapy management, leading to an overall reduction in the morbidity and mortality associated with HIV infection [1]–[5].

In recent years, there has been a reduction in the magnitude of the impact that opportunistic diseases have had on morbidity and mortality in cART patients in Brazil, although there has been an increase in the incidence of complications unrelated to infection by HIV, such as cardiovascular events, impaired renal function, liver disease and neoplasia [2], [6]–[12]. These complications occur in the context of both long-term exposure to cART and the rapid demographic transition that is underway in Brazil, which has resulted in an increase in the number of HIV-infected people over the age of 50 [13].

The impact of the Brazilian policy has been the object of nationwide studies, mostly focused on mortality [9], [10], [14], [15], the impact on the health system [1], [5], [16] and HIV resistance surveillance [17], [18], as well as small studies with limited representativeness, evaluating the clinical aspects of HIV infection [8], [11], [19] and the use of cART [20]–[24]. However, to date, there have been no nationwide longitudinal studies providing a broader picture of the Brazilian HIV epidemic and the results of the national HIV policy.

Furthermore, recently, important changes have occurred in the use of cART therapy due to the proven efficacy of these drugs to reduce the risk of acquiring and transmitting HIV infection, as well as increased evidence that early initiation of cART has significant health benefits [25]–[30]. This new context has fuelled the debate regarding the feasibility of achieving effective control over the HIV/AIDS epidemic in the near future [31].

These circumstances increase the relevance of studies assessing the various dimensions of access to diagnosis and treatment of HIV infections; the efficacy and effectiveness of such treatment; and the impact of this treatment on morbidity, mortality, and QOL. Therefore, the HIV-Brazil Cohort Study was established to gather further knowledge on the context of the Brazilian epidemic and to generate scientific evidence to make informed decisions regarding the planning and implementation of public policies, taking into consideration the regional disparities, as well as the specificities of the Brazilian population and of the national health care system [32]. The aim of this paper is to present the design and scope of the HIV-Brazil Cohort Study and the profile and initial cART of enrolled patients.

## Methods

### Cohort Sites

**Figure 1:**
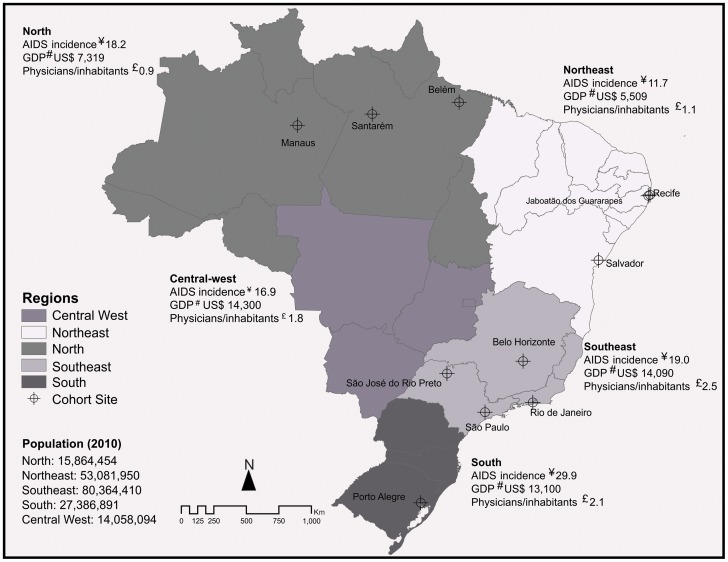
Characteristics of the regions and sites in the HIV-Brazil Cohort Study. Notes: ¥ Means per 100 000 population (2006 to 2010); ^#^Per capita gross domestic product (2010); £ number of physicians per 1000 inhabitants (2010). Source: Ministry of Health/DATASUS (Information Technology Department of the Brazilian National Health Care System) *and the Brazilian Institute of Geography and Statistics.*

There are 13 sites participating in the study, involving 26 public health facilities in 11 cities across four of the five regions of the country (Figure 1). The facilities were selected based on convenience, by region and city of location, the availability of information on the clinical follow-up and use of cART, and the existing infrastructure to conduct studies of this nature. The cities in which these facilities are located (Table 1) were chosen because they are representative of the diversity of the epidemiological profile of AIDS in Brazil [33] and account for 28% of all AIDS cases diagnosed in the country. Information regarding epidemiological surveillance derived by the Ministry of Health (Table 1) shows that, in 6 of the 11 cities, the incidence of AIDS cases is progressively increasing, having risen by an average of 30.7% from 2001 to 2010; in 7 of the cities, heterosexual transmission is the predominant route of HIV infection (in >60% of cases); and 6 of the cities are located in lower-income regions of the country (North or Northeast).

**Table 1:** Characteristics of the AIDS epidemic, sites involved and patient-selection process in the HIV-Brazil Cohort Study.

	Features of Aids,by City					Characteristics ofthe centers										Participation inthe Cohort	
	Incidence		Route ofHIV infection^c^			Patients		Services offered								Inclusion	
REGION/City/Site	Rate^a^	Increase^b^ (%)	Heterosexualcontact	Othersexualcontact	IDU	N per center d	% ofMunicipality e	Diagnostic	Home care	Hospitalization	In-houselaboratory	Pharmacy	P./Ph.^f^ ratio	Intervalbetweenfollow-up visits(months)	Absenteeoutreach	Phase	Criteria
**NORTH**																	
**Manaus**	42.1	68.1	67.8	25	2.7												
Tropical Medicine Foundation						5249	91–100	Yes	No	Yes	Yes	Yes	404	3	No	1st	Universal^g^
**Belém**	35.0	24.6	65.1	26.8	4.9												
UREDIPE						3950	51–90	Yes	Yes	No	No	Yes	263	3	No	1st	Universal^g^
**Santarém**	15.1	11.0	52.5	28.8	–												
Municipal STF						725	91–100	Yes	Yes	No	No	Yes	145	3	Yes	1st	Universal^g^
**NORTHEAST**																	
**Recife**	28.1	0.1	67.7	28.3	1.6												
UFPE						1300	≤20	No	No	Yes	Yes	Yes	217	4	No	2nd	Universal^g^
**Jaboatão de Guararapes**	25.1	3.9	70	25.5	1.6												
Municipal STF						519	51–90	Yes	No	No	No	Yes	260	3	Yes	2nd	Universal^g^
**Salvador**	23.5	14.6	59	29.8	6.9												
HUPES						1600	≤20	Yes	No	Yes	Yes	Yes	200	4	No	1st	Sample
CEDAP						10597	51–90	Yes	No	No	Yes	Yes	706	6	No	1st	Sample
**SOUTHEAST**																	
**Rio de Janeiro**	36.1	34924.9	60.5	35.2	2.5												
IPEC						3000	≤20	No	No	Yes	Yes	Yes	250	3	Yes	1st	Sample
**Belo Horizonte**	22.5	−11.1	53.4	39.9	4.5												
UFMG						3680	51–90	No	No	Yes	Yes	Yes	245	3	No	1st	Sample
**São Paulo**	25.3	−25.1	58	32.7	6.1												
CRT-SESSP						5000	≤20	Yes	Yes	Yes	Yes	Yes	77	3	Yes	1st	Universal^g^
Municipal Network h						34932	51–90	Yes	No	No	No	Yes	354	4	No	1st	Sample
**São José Rio Preto**	31.9	−31.1	70.7	22.6	5.4												
Municipal STF						1228	≤20	Yes	Yes	No	No	Yes	307	4	Yes	1st	Universal^g^
**SOUTH**																	
**Porto Alegre**	100.4	6.7	68.6	16	11.7												
Partenon Sanatorium						2281	≤20	Yes	No	No	No	Yes	456	6	Yes	1st	Universal^g^

Data Source: Features of AIDS by site: Data of the municipalities where the sites belonging to the study, with information from the National Epidemiological Surveillance System are; Characteristics of the centers: self-administered questionnaire by the manager of the service within the HIV- Brazil Cohort Study.

Abbreviations: Het., heterosexual; IDU, injection drug use; P./Ph., patient/physician; UREDIPE, *Unidade de Referência Especializada em Doenças Infecciosas e Parasitárias Especiais* (Referral Center Specializing in Specific Infectious and Parasitic Diseases); STF, specialized treatment facility; UFPE, *Universidade Federal de Pernambuco* (Federal University of Pernambuco); HUPES, *Hospital Universitário Professor Edgard Santos* (Professor Edgard Santos University Hospital); CEDAP, *Centro Estadual Especializado em Diagnóstico, Assistência e Pesquisa* (State Center Specializing in Diagnosis, Treatment and Research); IPEC, Instituto Evandro Chagas (Evandro Chagas Institute); UFMG, *Universidade Federal de Minas Gerais* (Federal University of Minas Gerais); CRT-SESSP, *Centro de Referência e Treinamento em DST e AIDS, Secretaria de Estado da Saúde de São Paulo* (São Paulo State Department of Health STD/AIDS Referral and Training Center).

a Per 100 000 population (2006–2010).

b 2001–2005 vs. 2006–2010.

c In relation to the total number of cases reported between 2006 and 2010.

d Patients alive at follow-up in the services, including use and non-use of cART.

e Proportion of the AIDS cases identified in the municipalities served by the facility.

f Patients/Infectious disease specialists or general clinicians ratio.

g Patients who meet the inclusion criteria between the total number of patients in the clinic follow-up sites.

h Fourteen health care facilities affiliated with the City of São Paulo municipality.

The number of patients under treatment at the participating sites ranged from 519 (at the Jaboatão de Guararapes Municipal Specialised Outreach Clinic) to 34,932 (within the São Paulo Municipal Network), with 7 of the 13 sites being responsible for treating more than half of the AIDS cases identified in their municipalities. Across the 13 sites, the mean ratio of outpatients to infectious disease specialists or general clinicians is 299∶1 (SD 158.8; range 77–706). Among the patients on cART, the interval between clinical consultations has ranged from 3 to 6 months, and 6 of the 13 sites have adopted outreach strategies for patients who miss appointments (Table 1).

At most of the sites (Table 1), the health care infrastructure includes other services, such as centers for the diagnosis of HIV infection (at all 13 sites), pharmacies (also at all 13 sites) and laboratories for monitoring HIV infection (at 7 sites), as well as inpatient units (at 6 sites) and home care services (at 4 sites).

### Eligibility and Inclusion Criteria

As of November 2012, 6,109 HIV-infected adults (≥18 years of age) had been enrolled in the cohort. All the included individuals had initiated antiretroviral treatment on or after January 1, 2003 (median year 2007; IQR 2004–2008), and had had at least one clinical follow-up appointment after treatment initiation. The inclusion occurred in two phases. Between 2009 and 2011, patients who had been started on cART between January 2003 and December 2010 were enrolled, whereas in the second phase, patients who had been started on cART on or after 1 January 2011 were enrolled. The first phase was aimed at observing events of interest that had occurred before the patients had been included in the cohort (retrospective phase), and the second phase was aimed at observing such events as they occurred, over the course of follow-up performed as routine clinical outpatient services (prospective phase).

Table 1 shows the sites involved in each phase and the procedures used in the selection of patients. At 8 of the 13 sites, eligible patients were identified from the total number of patients in clinical follow-up at each site, via an electronic cART dispensing system, and all such patients were included. At the 5 remaining sites, a non-probabilistic sample was used, with the selection procedures differing from site to site, depending on the features of the systems used for registering the cART dispensation. To identify possible discrepancies, the characteristics of the non-probabilistic samples at each site were compared with those of patients in the cohort already established for the region in which the site is located. For this comparison, patients included in the non-probabilistic samples were excluded from the set of individuals in the regional cohort. Similarities in terms of gender, age and lowest recorded CD4^+^ T-cell count were observed. The principal differences observed were that the proportion of females was greater in the non-probabilistic sample of the São Paulo Municipal Network than in the patients of the southeastern cohort (36.4% vs. 31.8%) and that the mean CD4^+^ T-cell count was higher in the non-probability sample of the Federal University of Minas Gerais than in the patients of the southeastern cohort (204.2 cells/mm^3^ vs. 174.2 cells/mm^3^).

### Data Sources and Outcomes

The cohort data were obtained as part of the routine clinical care provided at the health service centers included (routine-care based cohort) and were abstracted from the patients’ clinical records by trained abstractors onto standardized forms. These clinical records were reviewed at intervals not exceeding six months to investigate events recorded during the routine clinical follow-up visits performed within each period. To determine the consistency and completeness of the information, the forms were checked at the cohort coordination level. Approximately 5% of the forms were crosschecked against the information in the clinical records. From 2012 on, information has been collected using an online system developed on the Research Electronic Data Capture platform [34].

Information on QOL started being collected in 2011, using the World Health Organization self-report questionnaire (QOL Instrument-HIV, brief version) validated for use in Brazil [35]. The questionnaire is applied at follow-up initiation and every 12 months after the initiation of cART. Information about the treatment facilities, as well as the appropriateness and quality of the participant’s enrollment and data collection procedures, are obtained periodically through an electronic form completed at the sites.

The main outcomes of interest are the occurrence of diseases related or unrelated to HIV; suppression of viral replication (viral load <400 copies/ml [2003 to 2006] and <50 copies/ml [after 2007]); antiretroviral treatment modifications (initial or subsequent regimen); treatment failure (virological, clinical or immunological failure); adverse events; and death. Clinical and epidemiological data were collected for the entire clinical follow-up period, including socio-demographic data; HIV transmission category; use of illicit drugs, alcohol and tobacco; individual or family history of metabolic disorders, hypertension or cardiovascular disease; AIDS-related and non-AIDS-related manifestations; initial and subsequent cART regimens used for prophylaxis and treatment; occurrence of adverse events related to the use of cART, prophylaxis for opportunistic diseases and vaccines; CD4, viral load and genotyping results, as well as safety laboratory tests for cART monitoring; and mortality.

To enhance the level of completeness, missing data–notably, demographic data, CD4^+^ T-cell counts, viral load and mortality data–were systematically checked across the national information systems.

### Censoring Criteria

Individuals were censored in case of loss of clinical follow-up, relocation for follow-up in another clinical setting or death. To check the status of patients in the cohort, those categorized as lost to clinical follow-up were traced in the national information systems managed by the Brazilian National Ministry of Health: the National CD4^+^/CD8^+^ T Lymphocyte Count and Viral Load Network Laboratory Test Control System (for CD4^+^ T-cell counts and viral loads); the National System for the Logistic Control of Medications (for information regarding the dispensing of cART); and the National Mortality Information System (for mortality data). The screening was conducted annually, through electronic matching, checking for perfect combinations of key fields (patient’s name, mother’s name and date of birth). Patients not located through this procedure were then investigated using an alphabetically sorted list.

Loss of follow-up was defined as an absence of patient contact with the health care center for more than 12 months (i.e., no consultations, no CD4 or viral load exams performed, no records related to ARV refills in the clinical records) and the non-identification of the patient in the national information systems, as described above, up to July 2011. Patients were classified as having been relocated for follow-up in another clinical setting when relocations were noted in the clinical record or when the patients were identified in the information systems as performing their CD4^+^ T-cell count, viral load determination or ART dispensation elsewhere after the date of the last entry in the clinical record. AIDS-related deaths were defined as those in which the official record listed AIDS as being the underlying cause of death.

### Availability of Data

Proposals from external researchers to use our data are encouraged and welcomed, and requests will be analyzed by the Cohort Steering Committee that is composed of the Cohort Principal Investigators. The use of data pertaining to the individual sites is restricted to the sites themselves. In all circumstances, the confidentiality of each participant’s related data must be preserved.

### Ethics Statement

This protocol was approved by the Institutional Review Boards (IRB) of the participating sites (Comitê de Ética em Pesquisa da Secretaria Municipal de Saúde de São Paulo, Comitê de Ética do Centro de Referência e Treinamento DST/Aids, Comitê de Ética em Pesquisa do Instituto de Pesquisa Clinica Evandro Chagas da Fundação Oswaldo Cruz, Comitê de Ética na Pesquisa em Saúde da Escola de Saúde Pública da Secretaria de Estado da Saúde do Rio Grande do Sul, Comitê de Ética em Pesquisa da Maternidade Climério de Oliveira da Universidade Federal da Bahia and Comissão de Ética para Análise de Projetos de Pesquisa do Hospital das Clínicas e da Faculdade de Medicina da Universidade de São Paulo), according to the Brazilian regulation for research with human subjects. In the first phase, the IRB waived the requirement for written informed consent and requested the confidentiality of the individual’s data, which was ensured at all stages of the project. In the second phase, all participants provided written consent for participation in the study.

## Results

### Cohort Profile and Follow-up

The socio-demographic, clinical and immunological profiles of the 5,061 patients enrolled during the first phase of the study (those started on cART between 2003 and 2010) are shown in Table 2. There was a predominance of men (male/female ratio 1.7∶1), and the mean age at enrollment was 36.9 years (SD 9.9 years); 2,792 (55.2%) patients had been infected with HIV via heterosexual contact, 1,193 (23.6%) were in the homosexual/bisexual exposure category, and 2,300 (45.4%) were receiving cART at a treatment facility in the Southeast region.

**Table 2:** Characteristics of the patients and AIDS cases reported to the Brazilian Ministry of Health.

Variable	2003–2010			
	HIV-Brazil Cohort		Reported Cases of AIDS^a^	
	*(N)*	(%)	*(N)*	(%)
**Brazil (nationwide)**	5061	100.0	156391	100.0
**Region**				
North	1366	27.0	13159	8.4
Northeast	576	11.4	29448	18.8
Southeast	2300	45.4	74277	47.9
South	819	16.2	39505	25.3
No data available	–	–	2	0,0
**Gender**				
Male	3208	63.4	95952	61.4
Female	1853	36.6	60425	38.6
No data available	–	–	14	0.0
**Male/female ratio**	1.7	–	1.7	1.6
**Age group (years)**				
18 to 25	543	10.7	17708	11.3
26 to 30	925	18.3	25289	16.2
31 to 35	1030	20.4	28067	17.9
36 to 40	915	18.1	26240	16.8
41 to 45	711	14.0	22231	14.2
46 to 50	460	9.1	15588	10.0
51 to 90	475	9.4	21241	13.6
No data available	2	0.0	27	0.0
**Age (in years), mean (SD)**	36.9 (9.9)		37.9 (10.8)	
**Exposure Category**				
Heterosexual transmission	2792	55.2	66165	60.0
Homosexual transmission	859	17.0	14408	13.1
Bisexual transmission	334	6.6	6074	5.5
Unspecified sexual transmission^b^	203	4.0	–	–
Injection drug use	183	3.6	5838	5.3
Transfusion of blood or blood products	87	1.7	117	0.1
Vertical transmission	18	0.4	348	0.3
No data available	585	11.6	17323	15.7
**Pre-cART use of cocaine** b				
No	4318	85.3	–	–
Yes	743	14.7	–	–
**Lowest CD4^+^ T-cell count (cells/mm^3^)** b				
>350	241	4.8	–	–
200 ⊣ 350	1587	31.4	–	–
≤200	2704	53.4	–	–
No data available	529	10.5	–	–
**Nadir CD4^+^ T-cell count (cells/mm^3^), mean (SD)** b	177.5 (121.7)		–	–
**Clinical manifestation at the initiation of cART** b				
None	2062	40.7	–	–
Signs and symptoms	1620	32.0	–	–
Associated diseases	1379	27.2	–	–
**Initial regimen, by drug class** b				
2NRTIs+1NNRTI	3247	64.2	–	–
2NRTIs+PI/r	1220	24.1	–	–
2NRTIs+PI	511	10.1	–	–
Other	83	1.6	–	–
**Initial regimen, by drug** b				
AZT+3TC+EFZ	2436	48.1	–	–
AZT+3TC+LPV/r	724	14.3	–	–
AZT+3TC+ATV/r	232	4.6	–	–
Other	1663	32.6	–	–

Abbreviations: NRTI, nucleoside analogue reverse transcriptase inhibitor; NNRTI, non-nucleoside analogue reverse transcriptase inhibitor; PI/r, protease inhibitor, with adjuvant ritonavir; AZT, zidovudine; 3TC, lamivudine; EFZ, efavirenz; LPV/r, lopinavir/ritonavir; ATV/r, atazanavir/ritonavir.

a In individuals over 18 years of age.

b Data not included on AIDS case reporting form.

**Figure 2:**
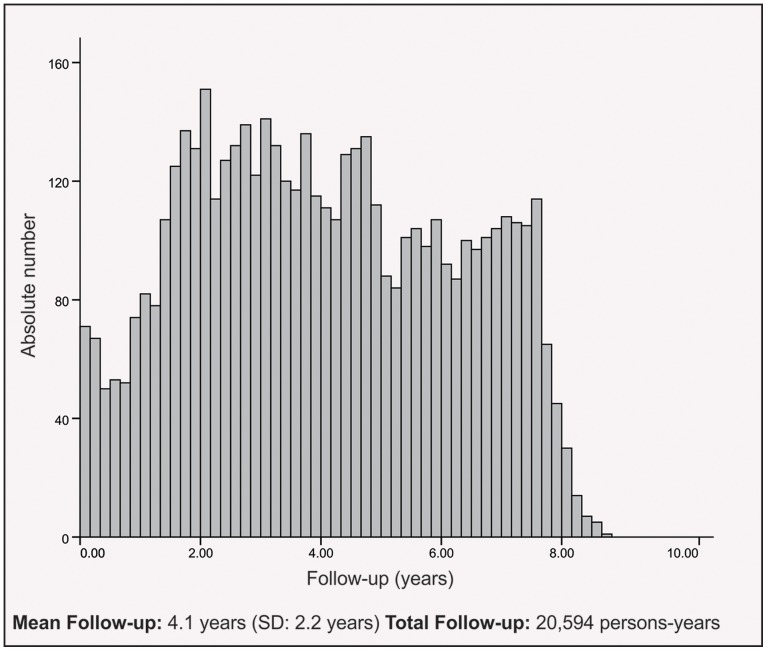
Patients included in the HIV-Brazil Cohort Study by follow-up time.

As shown in Figure 2, the median duration of follow-up was 4.1 years (IQR 2.2–5.9 years; maximum 8.8 years). During a period of 8.8 years, 206 (4.1% of the 5,061 cohort patients) were lost to follow-up, 345 (6.8%) were censored due to transfer from the health care centers where they were being followed in the cohort, and 287 (5.7%) had died. Therefore, the retention rate was 83.4%, with 20,593.9 person-years (PY) of observation.

The largest proportion of patient loss (104, 50.4%) was observed in the first year of cohort follow-up, with reductions over time. The loss to follow-up rate ranged from 19.8 per 1,000 PY in the first 12 months to 1.1 per 1,000 PY after the sixth year of observation, with an overall loss rate of 10.1 per 1,000 PY. The greatest risk of loss (Table 3) was observed for the health care centers located in the less developed regions (21.3 per 1,000 PY [North] and 10.9 per 1,000 PY [Northeast]), among young patients (18 to 25 years [14.0 per 1,000 PY] and 26 to 30 years [15.6 per 1,000 PY]), bisexuals (13.8 per 1,000 PY), those with no clinical manifestations after cART initiation (12.6 per 1,000 PY) and those with no history of cocaine use before cART initiation (12.1 per 1,000 PY).

**Table 3:** Descriptive analysis of loss of follow-up^a^ in the HIV-Brazil Cohort Study.

Characteristics	HIV-Brazil Cohort	Loss of follow-up		95% CI of the proportion		Rate of follow up loss (1000 PY)
	*N*	*N*	*(%)*	*Minimum*	*Maximum*	
**Gender**						
Male	3208	147	4.6	3.9	5.4	11.4
Female	1853	59	3.2	2.5	4.1	7.7
**Sexual Orientation** b						
Homosexual	906	20	2.2	1.4	3.4	5.3
Bisexual	374	21	5.6	3.7	8.4	13.8
Heterosexual	2983	113	3.8	3.2	4.5	9.1
**Age group (years)** b						
18 to 25	543	30	5.5	3.9	7.8	14.0
26 to 30	925	56	6.1	4.7	7.8	15.6
31 to 35	1030	43	4.2	3.1	5.6	10.1
36 to 40	915	27	3.0	2.0	4.3	6.9
41 to 45	711	30	4.2	3.0	6.0	10.3
46 to 50	460	6	1.3	0.6	2.8	3.2
51 to 90	475	12	2.5	1.5	4.4	6.3
**Region**						
North	1366	105	7.7	6.4	9.2	21.3
Northeast	576	25	4.3	3.0	6.3	10.9
Southeast	2300	63	2.7	2.1	3.5	5.9
South	819	13	1.6	1.3	3.2	4.9
**Pre-cART clinical manifestation**						
None	2062	84	4.1	3.3	5.0	10.0
Signs and symptoms	1620	70	4.3	3.4	5.4	10.7
Associated disease	1379	52	3.8	2.9	5.0	9.2
**Post-cART clinical manifestation**						
None	2762	131	4.7	4.0	5.6	12.6
Signs and symptoms	1311	38	2.9	2.1	4.0	6.6
Associated disease	988	37	3.7	2.7	5.1	8.2
**Pre-cART use of cocaine**						
Yes	4318	170	3.9	3.4	4.6	9.6
No	743	36	4.8	3.5	6.6	12.1
**Lowest CD4+ T-cell count (mm^3^)** b						
>350	241	10	4.1	2.2	7.4	8.8
200 ⊣ 350	1587	69	4.3	3.4	5.4	10.7
≤200	2704	97	3.6	2.9	4.3	9.9

Abbreviation: PY, person-years.

a Maximum follow-up time of 8.8 years.

b Not included are 798 individuals with unknown transmission categories, 2 with unknown ages and 529 without a CD4–T exam prior to cART initiation.

### Characteristic of the Initial Treatment

Information on the pre-cART immunological status is available for 89.6% of the patients, who presented with a mean and median nadir CD4^+^ T-cell count of 177.5 cells/mm^3^ (SD 121.7 cells/mm^3^) and 169.0 cells/mm^3^ (IQR 75.0–250.0 cells/mm^3^), respectively. These values remained stable over the years evaluated (β<−0.001; *p*<0.99). The majority of patients (53.4%) initiated cART with a nadir CD4^+^ T-cell count ≤200 cells/mm^3^, and the CD4^+^ T-cell count at cART initiation was ≤350 cells/mm^3^ in 84.8% of the sample.

The initial treatment regimens prescribed were in line with the current recommendations of the Brazilian National Ministry of Health [36], and 28 cases (of the 5,061 cases) were identified in which inappropriate regimens were prescribed. Among the initial treatments, regimens involving non-nucleoside analogue reverse transcriptase inhibitors, boosted protease inhibitors and protease inhibitors without booster were prescribed for 64.2%, 24.1% and 10.1% of the patients, respectively. In 48.1% of the 5,061 cases, the first-line treatment regimen prescribed was zidovudine+lamivudine+efavirenz (AZT+3TC+EFZ), whereas AZT+3TC+lopinavir/ritonavir (AZT+3TC+LPV/r) and AZT+3TC+atazanavir/ritonavir (AZT+3TC+ATV/r) were used in 14.3% and 4.6%, respectively. In the 2009–2010 period, there was an increase in the number of cases in which tenofovir was prescribed, (11.6% in 2010).

The median number of regimens used per patient was 1 (IQR 1–2; mean 1.8), and the mean duration of the first-line regimens was 30.4 months (95% CI 29.7–31.2). The number of individuals achieving viral suppression (VL <400 copies/ml [2003 to 2006] and <50 copies/ml [after 2007]) within the first 12 months after cART initiation was 77.4% (95% CI 76.1–78.6). During the 6.5 years of observation, 88.2% (95% CI 87.2–89.1) of the patients achieved viral suppression at some point after cART initiation. The lowest rates of viral suppression in the first year of cART were observed in individuals aged between 18 and 29 years (66.2%), in transmission category injection drug use (73.7%), with cocaine use before treatment (74.1%), in service areas with lower economic development (North and Northeast regions, 61.3% 73.3%) and showing no adverse ARV events (85.2%).

Nearly half (45.4%) of the patients presented with HIV-related clinical manifestations after starting cART, and the most frequent were oral candidiasis and tuberculosis. The AIDS mortality rate for the period evaluated was 13.9 per 1,000 PY.

## Discussion

The HIV-Brazil Cohort Study, with a long follow-up period and a significant number of observations, is an important asset to increase the availability of data on the countrywide outcomes of the National AIDS Program related to cART in public health care services. A special feature of our cohort is that HIV-infected individuals in Brazil have been exposed to a broad array of antiretroviral drugs–21 antiretroviral drugs of all classes (including 10 generic drugs) used in first-, second- and third-line treatment regimens [4]–for a longer period than patients from other resource-limited settings. Consequently, the results obtained from this cohort might be predictive of the effects that the longer-term use of cART will have in other middle- or low-income countries, where the regimens currently in use in Brazil have yet to be widely applied.

The characteristics of the HIV-Brazil Cohort Study facilitates analyses of the short-, medium- and long-term impact of using antiretroviral drugs in the context of the routine care of patients at publically funded health care centers, providing complementary evidence to that obtained through clinical efficacy trials. The evidence obtained from the analyses performed in this cohort will enable field evaluations and further improvement of the national public health policies [37].

The socio-demographic profile of the HIV-Brazil Cohort Study is similar to that represented by the cases of AIDS in adults reported to the Brazilian National Ministry of Health between 2003 and 2010 (Table 2). The two groups are also comparable in terms of the HIV transmission category and region of origin, although, proportionally, the HIV-Brazil Cohort Study included fewer individuals treated in the southern region of the country, resulting in a smaller representation of injection drug users.

The quality assessment of the data collected showed a satisfactory degree of completeness of the information for essential variables (Table 2). A lack of CD4+ T-cell counts prior to the initiation of cART was observed in 1 of 10 patients and was probably related to clinical decisions not to request this testing if the patient had a concomitant opportunistic infection at the time of cART initiation or during hospitalization. The epidemiological characteristics of individuals without baseline information on CD4^+^ T-cell count, such as gender, age and transmission category, did not differ (p<0.05) from the other patients included in the cohort. Although the analysis of outcomes directly related to the CD4+ T-cell count showed few significant variations when excluding individuals without baseline information on CD4+ T-cell count, the mortality rate rose from 13.9 to 14.2 per 1,000 PY.

An important aspect to be highlighted is the relatively low rate of loss of follow-up, similar to what is observed in high-income countries and in the middle- and low-income countries reporting the lowest rates of loss of follow up [38], [39]. The greater accuracy of this information was obtained through the linkage with national health information systems databases. These databases have high rates of coverage, gathering data from all HIV-infected patients linked to care and receiving ART nationwide, as well as the overall mortality data in the country [14]. Consequently, patients classified as lost to follow-up in the cohort have a high possibility of still being alive even without appearing for the clinical follow-up or are using cART from some other health service in the country.

The analysis presented in this article aims to outline the scope and potential use of the information derived from the HIV-Brazil Cohort Study. First-line non-nucleoside reverse-transcriptase-based regimens were the most prescribed first-line cART in Brazil and resulted in high levels of viral suppression in the first year of treatment, comparable to single-site observational studies [20], [23], [24], [40]. This finding has reinforced the assumption that the use of cART in Brazilian health services has achieved results similar to other low- and middle-income countries [41]–[42].

The majority of patients presented with severe immunodeficiency (CD4^+^ T-cell counts ≤200 cells/mm^3^) or AIDS-related diseases at cART initiation. This finding was higher than the results of previously reported studies that reviewed the clinical and immunological status at the time of arrival to the health services for initial clinical follow-up in Brazil [14]. Consequently, a higher proportion of late-onset cART in HIV-Brazil Cohort Study might have been caused by the loss of clinical follow-up before the initial prescription of cART.

It is also noteworthy that the mean CD4+ T-cell count at the beginning of cART did not show a trend of increase over time, indicating the urgent need for increased access to earlier HIV diagnosis and linkage to care countrywide, considering the fact that Brazil has a concentrated epidemic in social sectors with a high degree of stigma and a pattern of service use that is more restricted than in the general population [32], [33].

Another important aspect is the fact that the worst results were observed in regions with the lowest economic development levels in the country, indicating that the reduced ability of these regions to obtain optimal results from the therapies available for AIDS may lead to an increase in the health inequalities existing in the country through increase HIV incidence and HIV-associated mortality.

The study design, characteristics of patients included and the initiation of cART presented in this paper show the scope of investigation conducted by the HIV-Brazil Cohort Study, which is the main initiative to analyze the effects of cART use in public health services in the country. Thus, the results shown herein and those yet to be produced can contribute to advancing the knowledge generated from clinical trials, particularly considering the context of medium- and low-income countries, which in recent years have greatly expanded their programs to access cART. This is important in the formulation of better health policies for the needs of people living with HIV in resource-constrained situations.
