# Correction: The HIV-Brazil Cohort Study: Design, Methods and Participant Characteristics

**DOI:** 10.1371/journal.pone.0104119

**Published:** 2014-07-25

**Authors:** The PLOS ONE Staff

There are multiple errors in the article as described below:

1. The full name for Dr. Valdiléa Veloso is Dr. Valdiléa G. Veloso.
2. The correct name for Dr. Alex Jones Flores Cassanote is Dr. Alex Jones Flores Cassenote.
3. The PDF version does not contain a full list of references. Please refer to the online version for the full list of references.
